# CoBRA: Containerized Bioinformatics Workflow for Reproducible ChIP/ATAC-seq Analysis

**DOI:** 10.1016/j.gpb.2020.11.007

**Published:** 2021-07-18

**Authors:** Xintao Qiu, Avery S. Feit, Ariel Feiglin, Yingtian Xie, Nikolas Kesten, Len Taing, Joseph Perkins, Shengqing Gu, Yihao Li, Paloma Cejas, Ningxuan Zhou, Rinath Jeselsohn, Myles Brown, X. Shirley Liu, Henry W. Long

**Affiliations:** 1 Center for Functional Cancer Epigenetics, Dana-Farber Cancer Institute, Boston, MA 02215, USA; 2 Department of Medical Oncology, Dana-Farber Cancer Institute, Harvard Medical School, Boston, MA 02215, USA; 3 Albert Einstein College of Medicine, Bronx, NY 10461, USA; 4 Department of Biomedical Informatics, Harvard Medical School, Boston, MA 02215, USA; 5 Department of Data Sciences, Dana Farber Cancer Institute, Harvard T.H. Chan School of Public Health, Boston, MA 02215, USA

**Keywords:** ChIP-seq, ATAC-seq, Snakemake, Docker, Workflow

## Abstract

Chromatin immunoprecipitation sequencing (ChIP-seq) and the Assay for Transposase-Accessible Chromatin with high-throughput sequencing (ATAC-seq) have become essential technologies to effectively measure protein–DNA interactions and chromatin accessibility. However, there is a need for a scalable and reproducible pipeline that incorporates proper normalization between samples, correction of copy number variations, and integration of new downstream analysis tools. Here we present Containerized Bioinformatics workflow for Reproducible ChIP/ATAC-seq Analysis (CoBRA), a modularized computational workflow which quantifies ChIP-seq and ATAC-seq peak regions and performs unsupervised and supervised analyses. CoBRA provides a comprehensive state-of-the-art ChIP-seq and ATAC-seq analysis pipeline that can be used by scientists with limited computational experience. This enables researchers to gain rapid insight into protein–DNA interactions and chromatin accessibility through sample clustering, differential peak calling, motif enrichment, comparison of sites to a reference database, and pathway analysis. CoBRA is publicly available online at https://bitbucket.org/cfce/cobra

## Introduction

**Figure 1:**
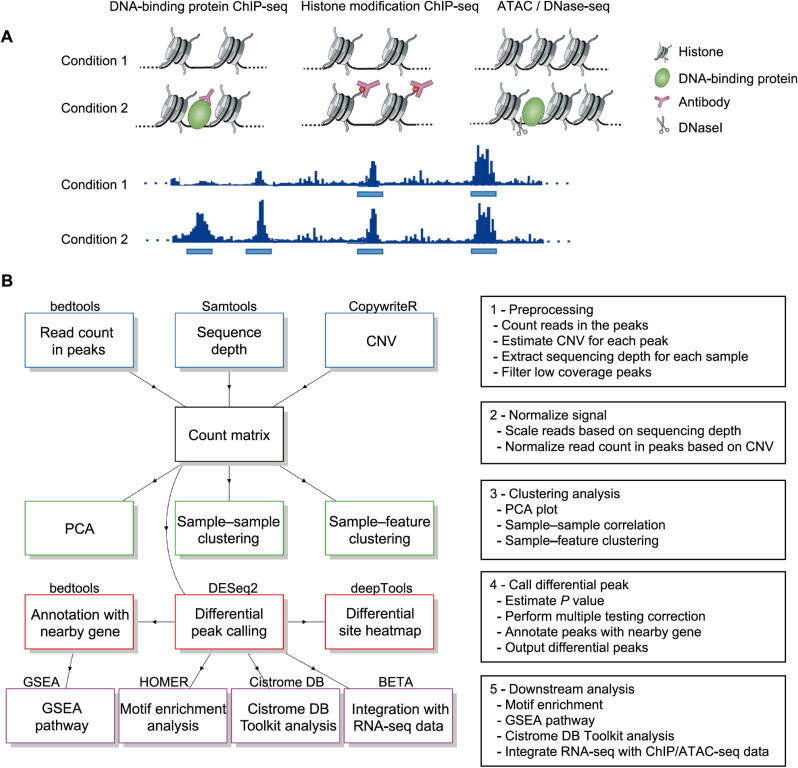
**Overview of CoBRA. A.** Biological motivation of CoBRA. Comparisons between ChIP-seq and ATAC-seq peaks in well-designed experiments can provide insight into differences in protein occupancy, histone marks, and chromatin accessibility. Bottom diagrams show different types of peak comparisons, the light blue bars represent significant peaks called by MACS2. **B.** Overview of the workflow performed by CoBRA. Read counts are quantified and normalized for sequencing depth and CNV before clustering and differential peak calling analyses. The result of differential peak calling is used for downstream analyses, including motif enrichment analysis, GSEA pathway analysis, Cistrome DB Toolkit analysis, and BETA. CoBRA, Containerized Bioinformatics workflow for Reproducible ChIP/ATAC-seq Analysis; ChIP-seq, chromatin immunoprecipitation sequencing; ATAC-seq, Assay for Transposase-Accessible Chromatin with high-throughput sequencing; CNV, copy number variation; GSEA, Gene Set Enrichment Analysis; BETA, Binding and Expression Target Analysis; HOMER, Hypergeometric Optimization of Motif EnRichment; PCA, principal component analysis; Cistrome DB, Cistrome Data Browser.

Chromatin immunoprecipitation sequencing (ChIP-seq) and the Assay for Transposase-Accessible Chromatin with high-throughput sequencing (ATAC-seq) have become essential components of epigenetic analysis, which are employed extensively in the study of protein–DNA interactions and chromatin accessibility, respectively. ChIP-seq is a high‐throughput technology that provides unique insights into protein function by mapping genome-wide binding sites of DNA-associated proteins. ATAC-seq is a high-throughput technology that is imperative in the assessment of genome-wide chromatin accessibility. While numerous pipelines for analyzing ChIP‐seq and ATAC-seq data have been reported in the literature [1], [2], [3], [4], [5], [6], [7], [8], there remains a strong need for pipelines that can be run by users who have limited experience in utilizing computational biology tools. Comparisons between ChIP-seq and ATAC-seq experiments can provide insight into differences in protein occupancy, histone marks, and chromatin accessibility (Figure 1**A**). However, analysis pipelines currently available lack useful components necessary for such analyses. For example, there is a need for better normalization between samples, adjusting copy number variations (CNVs), applying newly developed downstream annotation tools such as Cistrome DB Toolkit [9], and integrating epigenetic data with RNA-seq data.

In this work, we developed a modularized computational workflow, Containerized Bioinformatics workflow for Reproducible ChIP/ATAC-seq Analysis (CoBRA). CoBRA can quantify ChIP-seq and ATAC-seq peak regions, and perform unsupervised and supervised analyses. It provides sample clustering, differential peak calling, motif enrichment and clustering, annotation of differential sites by a reference database, and pathway analysis. In addition, it also provides clear, high-quality visualizations for all results.

CoBRA uses Snakemake [10], a workflow management system to create the computational pipeline. Using the Snakemake system enables the reproducibility and scalability of CoBRA. This framework also allows for the addition or replacement of analysis tools, as well as for the parallelization of computationally intensive processes. To make CoBRA portable, the workflow and its software dependencies are available as a Docker container, which can be used on any machine with Docker installed. This includes local servers, high-performance clusters, and cloud-based machines. Docker automatically downloads all required software dependencies because the container encapsulates all of the supporting software and libraries, eliminating the possibility of conflicting dependencies.

CoBRA therefore provides solutions to challenges inherent to many bioinformatics workflows: it is portable, reproducible, scalable, and easy to use. It is open source (https://bitbucket.org/cfce/cobra), well documented online. Detailed step-by-step tutorials are included, which go through all three case studies presented in this paper (https://cfce-cobra.readthedocs.io). The combination of features enables researchers to gain rapid insight into protein–DNA interactions and chromatin accessibility with comprehensive state-of-the-art ChIP-seq and ATAC-seq analysis.

## Method

### Overall design

The CoBRA pipeline is implemented using the Snakemake workflow management system [10] and is described via a human-readable, Python-based language. This allows CoBRA to scale to server, cluster, grid, and cloud environments, without the need to modify the workflow. For ChIP-seq and ATAC-seq experiments, CoBRA provides both unsupervised and supervised analyses (Figure 1B). It does not include ChIP-seq and ATAC-seq quality control steps, as this is best handled within other specialized pipelines [11].

Furthermore, CoBRA is distributed as a Docker container, which can be used on any machine as long as Docker is installed. The container encapsulates all of the supporting software and libraries, eliminating the possibility of conflicting dependencies, and facilitating the installation of required software. With the built-in Snakemake reference rule, CoBRA automatically downloads all needed reference files, if they have not been downloaded before. Users specify analysis parameters in a simple human-readable configuration file (Figure S1A-C). A separate file contains metadata about the samples being analyzed (cell line, treatment, time point, etc.), as well as a specification of the differential comparisons to be performed by the pipeline. This metadata file is in CSV format and can be easily modified in any standard text editor or Excel. Example of input and output file structure is in Figure S2.

### Unsupervised analysis

The pipeline calculates the reads per kilobase per million mapped reads (RPKM) using bed files and bam files provided by the user to normalize based on sequencing depth and peak size. The RPKM table is filtered through the removal of sites that have low RPKM values across multiple samples. Quantile normalization (default), z-score, and log transformation are available options to normalize the count matrix. To visualize the similarities between samples in the experiment, sample–sample correlation, principal component analysis (PCA), and sample–feature plot are automatically generated by the pipeline.

The sample–sample correlation plot illustrates the similarity between all of the samples on a pairwise basis. It also provides the clustering result based on the Pearson correlation coefficient (*r*), where distance is defined as 1− r. The user can opt for using Spearman correlation, as well as selecting other distance methods (Euclidean, Manhattan, Canberra, binary, maximum, or Makowski) by simply changing the configuration file. The resulting correlation plot helps to determine whether the different sample types can be separated, *i.e.*, samples of different conditions are expected to be more dissimilar to each other than replicates within the same condition. User-provided metadata are used to automatically annotate samples in all unsupervised plots.

Subsequently, CoBRA produces a PCA plot depicting how samples are separated in the first two PCs (those with the largest variance) and samples are automatically color-coded by all user-provided annotations. The PCA plot helps the user to determine whether any patterns exist between the samples and whether outliers are present. Finally, CoBRA generates a sample–feature heatmap. The heatmap illustrates the clustering of samples based on correlation on the horizontal axis and clustering of peaks on the vertical axis. Peaks on the vertical axis can be clustered by hierarchical or *k*-means clustering. The sample–feature heatmap elucidates patterns of peaks across samples and identifies the clusters that are enriched in a subset of samples.

### Supervised analysis

A common question asked in epigenetic experiments is what are the differential sites (transcription factor binding, histone modification, and chromatin accessibility) between sample groups. Several tools currently available can be applied to analyze differential sites, most of which are derived from RNA-seq count analysis (DESeq2, edgeR, and Limma). However, there are differences between the RNA-seq and ChIP-seq count analyses. In RNA-seq experiments, most reads are in the exome, where read count can be normalized by the total number of reads mapped to all genes. In contrast, most ChIP-seq reads are outside of peaks. The fraction of reads in peaks (FRiP) score typically ranges from 1%–40% [11]. Reads in peaks are only a portion of total reads that have been sequenced. Therefore, all reads need to be normalized by the total number of uniquely mapped reads to account for sequence depth. CoBRA uses the bam file to calculate sequencing depth. It utilizes sequencing depth as a scale factor in differential peak calling by DESeq2 (although the user can specify reads in peaks for scaling if specifically required). This is an essential step in differential peak calling. The default scale factor utilized by DESeq2 to normalize the data is the total number of reads mapped to peaks, which can result in the calling of false positive differential peaks. Instead, using sequencing depth as the scale factor ensures that reads are normalized for experimental variation and not biological variation between samples.

Multiple comparisons can be done within a single run. For each comparison, the number of differential peaks for two adjusted *P* value cutoffs and two fold change (FC) cutoffs is displayed in a summary chart. Furthermore, the bigwig files are used to plot the peak intensity of the differential peaks in a heatmap using deepTools2 [12].

The differentially enriched regions from DESeq2 for each comparison are subsequently run through HOMER [13] for motif enrichment analysis. Motif enrichment analysis is a fundamental approach to look for transcription factor motifs that might be enriched in regions of interest. We use HOMER in the pipeline to look for known and *de novo* motifs that are enriched in the differential peak regions compared to GC matched, randomly-selected genome background. In addition, we utilize a motif clustering algorithm to organize various motifs by similarity, making the output easier to evaluate for distinct results. By mapping the peaks to the nearest genes, CoBRA uses Gene Set Enrichment Analysis (GSEA) pre-ranked analysis to investigate the pathways that are enriched and depleted for both upregulated and downregulated peaks.

The upregulated and downregulated sites are also automatically compared to a comprehensive database of ChIP/ATAC-seq and DNase l hypersensitive sites sequencing (DNase-seq) data [9], [14]. The Cistrome DB Toolkit analysis determines the most similar samples in terms of genomic intervals overlapping with the differential sites. The toolkit is particularly useful to identify the major transcription factors related to the differential perturbations. In addition, it can be useful in the identification of potential biological sources (cell line, cell type, and tissue type) of similarity to the regions of interest.

## Results

In order to illustrate the utility of CoBRA, we applied it to three case studies with components to showcase the different capabilities of our workflow. These include a glucocorticoid receptor (GR) ChIP-seq dataset from the ENCODE project, an H3K27ac ChIP-seq data from colon cancer cell lines, and an ATAC-seq experiment on HL-60 promyelocytes differentiating into macrophages. Each example demonstrates some key functions of the CoBRA pipeline.

Case studies

Example 1: *normalizing GR ChIP-seq data in a dose–response experiment*

**Figure 2:**
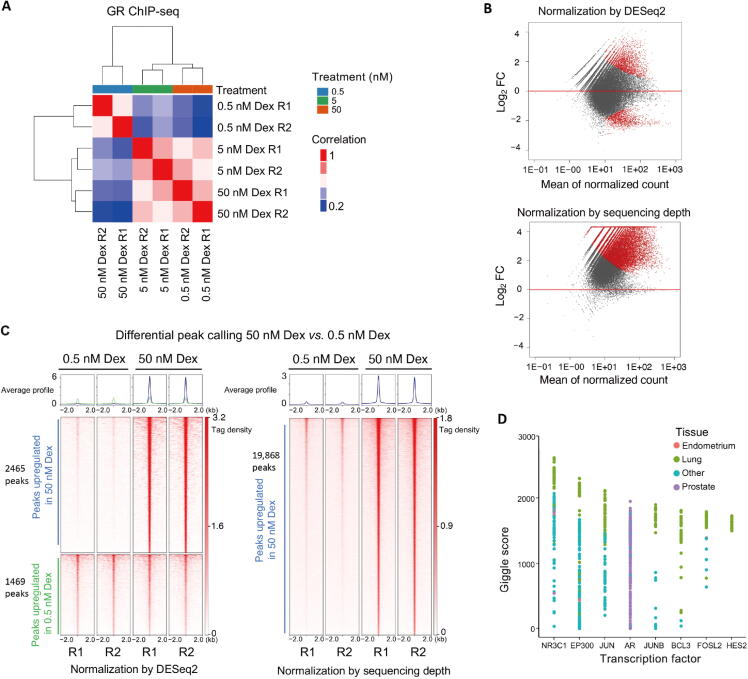
**Example of unsupervised and supervised analyses of differential GR binding in A549 cells. A.** Sample–sample heatmap depicting clustering and correlation between A549 cells treated with varying concentrations (0.5 nM, 5 nM, and 50 nM) of Dex in duplicates. **B.** Visualization of the differences in GR binding between the samples treated with 0.5 nM and 50 nM Dex, plotted using mean of the ChIP-seq peak intensities against Log_2_ FC of GR binding at the concentrations of 0.5 nM and 50 nM. This illustrates the change in the inferred differential GR binding profile following normalization using scaling factor determined by total reads in peaks (top) and sequencing depth (bottom). **C.** DeepTools heatmap illustrating differential peaks called by DESeq2 using default scaling factor by total reads in peaks (left) or using scaling factor determined by sequencing depth (right). A group of peaks at the bottom of the left panel exhibit similar binding intensity, however, they are considered downregulated in samples treated with 50 nM Dex, in the peak calling result with default DESeq2 setting. **D.** Cistrome DB Toolkit analysis result illustrating publicly available ChIP-seq datasets from Cistrome DB ranked by binding profile similarity to gained GR binding sites with Dex treatment. Dex, dexamethasone; GR, glucocorticoid receptor; FC, fold change.

We downloaded publicly available GR ChIP-seq data (GEO: GSE32465) from a lung adenocarcinoma cell line (A549) at 3 different concentrations of dexamethasone, a potent GR agonist. In an analysis of this dataset [15], it has been found that the number of GR binding sites increases with increasing dexamethasone concentration. In the experiment, samples were treated with 0.5 nM, 5 nM, or 50 nM dexamethasone. Using the unsupervised analysis in CoBRA, it is shown that the sample replicates cluster tightly together. Similarities and differences between samples are illustrated by the correlation between treatments *vs.* within treatment in the dendrogram at the top of sample–sample heatmap (Figure 2**A**), as well as the PCA plot (Figure S3A).

While unsupervised analyses are useful, the advantage of the CoBRA pipeline is its ability to accurately call differential peaks accounting for a variety of factors. We applied DESeq2 to assess the differences in peak binding for samples treated with 50 nM dexamethasone *vs.* samples treated with 0.5 nM dexamethasone. Utilizing the default scale factor method in DESeq2, which normalizes the data using the total number of reads in peaks, differential peaks are called (Figure 2B) where they are clearly not present (Figure 2C, left). A group of peaks (Figure 2C, bottom left) exhibit similar binding intensity. However, in the DESeq2 result, these peaks are considered downregulated in samples treated with 50 nM dexamethasone.

DESeq2 by default normalizes all samples by total reads in the read count table. In RNA-seq experiments, most reads are in the exome, where reads can be normalized by the total number of reads mapped to all genes. In contrast, in the GR ChIP-seq experiment, samples treated with 50 nM dexamethasone exhibit much more GR binding peaks (29,921 *vs.* 3397)and higher FRiP score (9.3 *vs.* 0.9) than samples treated with 0.5 nM dexamethasone. Therefore, the normalization method used in DESeq2 decreases the peak intensity in the samples treated with 0.5 nM dexamethasone because the FRiP scores are higher in the samples treated with 50 nM dexamethasone, resulting in the calling of false positive differential peaks (Figure 2C, right). In CoBRA, we use a scaling factor dependent on the sequencing depth of each sample. This eliminates the calling of false positive downregulated peaks that are called by DESeq2 using the default scaling factor (Figure 2B and C, right). Furthermore, more true differential gained peaks have been successfully identified with the scaling method in CoBRA.

An additional feature of CoBRA is that it automatically analyzes the differential peaks to provide additional insight into their origins and identify similar systems in the literature. In one analysis it determines the most similar ChIP-seq data that is available in Cistrome Data Browser (Cistrome DB; cistrome.org), a large, curated database of ChIP-seq and ATAC-seq data [14]. For the gained GR binding sites in the dexamethasone treatment, the result from the Cistrome DB Toolkit [9] clearly shows that the *NR3C1* ChIP-seq peaks in lung tissue is the most similar to gained GR binding sites in the Cistrome DB (Figure 2D). CoBRA provides a list of Gene Expression Omnibus (GEO) accession numbers corresponding to all ChIP-seq data with similarity to the differential peak set. Using these identifiers, ChIP-seq data of interest can be downloaded for further investigation from Cistrome DB [14]. While obviously correct in this simple case, this tool can provide unique insight into gained or lost sites, such as suggesting which transcription factor potentially binds to a differential peak set after a perturbation and identifying potentially similar cellular systems. In addition, CoBRA performs a *de novo* motif analysis on differential sites (Figure S3B), which can help to identify potential transcriptional regulators enriched in the differentially accessible chromatin elements. In this example, the top cluster has all hormone receptor motifs enriched in the upregulated peaks.

Example 2: *correcting CNVs in H3K27ac ChIP-seq data*

We further illustrate the advantages of CoBRA pipeline utilizing data from colorectal cancer cell lines. Microsatellite instable (MSI) and microsatellite stable (MSS) tumors are two classes used to characterize colorectal cancers. To analyze these cell lines, we selected six publicly available datasets from several experiments: three MSI samples and three MSS samples [16], [17], [18], [19], [20] (GEO: GSM1866974, GSM2265670, GSM1224664, GSM1890746, GSM2058027, and GSM1890746).

**Figure 3:**
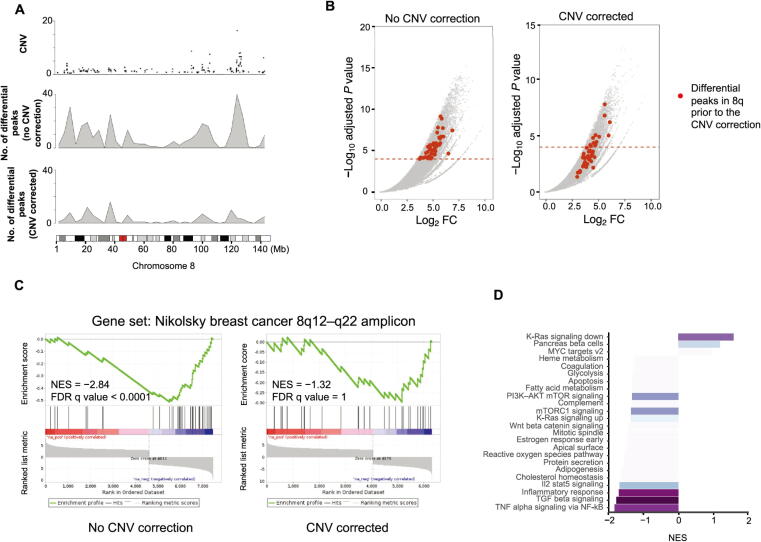
**Identification of differential sites with CNV correction. A.** Copy number distribution for an MSS sample on Chr8 (top). Distribution of differentially called peaks without (middle) and with (bottom) CNV correction between MSS and MSI cell lines. The chromosomal plot for Chr8 with genomic coordinate is provided at the very bottom and the red block in the middle indicates the centromere of Chr8. **B.** Differential peaks in Chr8q called with or without CNV correction. The x axis indicates the Log_2_ FC of H3K27ac signal difference between MSS and MSI cell lines and y axis indicates −log_10_ FDR-adjusted *P* value. Plot on the left shows the peak distribution without CNV correction, and plot on the right shows the peak distribution with CNV correction. Significantly differential (FDR-adjusted *P* ≤ 0.0001) peaks in Chr8q prior to CNV correction are highlighted in red. Dashed lines indicate the cutoff FDR adjusted *P* values for significance. **C.** Enrichment plot for NIKOLSKY_BREAST_CANCER_8Q12_Q22_AMPLICON gene set without (on the left) and with (on the right) CNV correction. **D.** Enrichment of the GSEA hallmark gene sets after CNV correction based on the differential peak ranking comparing MSS with MSI tumors. MSS, microsatellite stable; MSI, microsatellite instable; CNV, copy number variation; NES, normalized enrichment score; FDR, false discovery rate.

MSS tumors are one of the most highly mutated tumor types [21] and typically exhibit a high number of copy number alterations. Without correction, a differential peak caller will rank peak loci with high copy number gain in MSS as being the most differential compared to MSI. These genetic differences, while important, can obscure important epigenetic differences between MSI and MSS tumors. In order to observe differential peaks other than those called as a result of the presence of CNVs, CNV correction was conducted on all samples. For this example, the copy number was called using the ChIP-seq data itself with CopywriteR [22] but can also be done with QDNAseq [23] using the input control if available. Any other sources of CNV data can also be used when put in a standard igv format. This CNV correction alters the differential peaks called by DESeq2. In the case of the MSS *vs.* MSI comparison, many peaks at the q arm of chromosome 8 are called significantly differential (Figure 3**A**) but, following CNV correction, the number of differential peaks in this region significantly decreases (Figure 3B).

GSEA is performed on the ranked list of genes produced by CoBRA. Without CNV correction, GSEA can indicate greatest enrichment in gene sets solely related to amplification. As a result, it is challenging to assess the true epigenetic differences between these two colorectal cancer types. For instance, the gene set ‘NIKOLSKY_BREAST_CANCER_8Q12_Q22_AMPLICON’ includes genes with up-regulated expression in non-metastatic breast cancer tumors with amplification in the 8q22 region. Without correction for CNVs, this gene set is significantly enriched (Figure 3C). It is the 3th ranked gene set, with a normalized enrichment score of −2.84 and an adjusted *P* value < 0.0001. With CNV correction, this gene set is far less enriched (Figure 3C). It is the 468th ranked gene set and has a normalized enrichment score of −1.32 and an adjusted *P* value of 1, indicating that the enrichment is not statistically significant.

After CNV correction, GSEA of the hallmark gene sets shows that the MSI cell line exhibits enrichment in the following pathways: TNF∞ signaling via NF-κB, TGFβ signaling, and inflammatory response (Figure 3D). This is consistent with the literature [24], [25] in reference to colon cancer with MSS tumors exhibiting more inflammatory signaling.

Example 3: *unsupervised analysis of time series ATAC-seq data*

**Figure 4:**
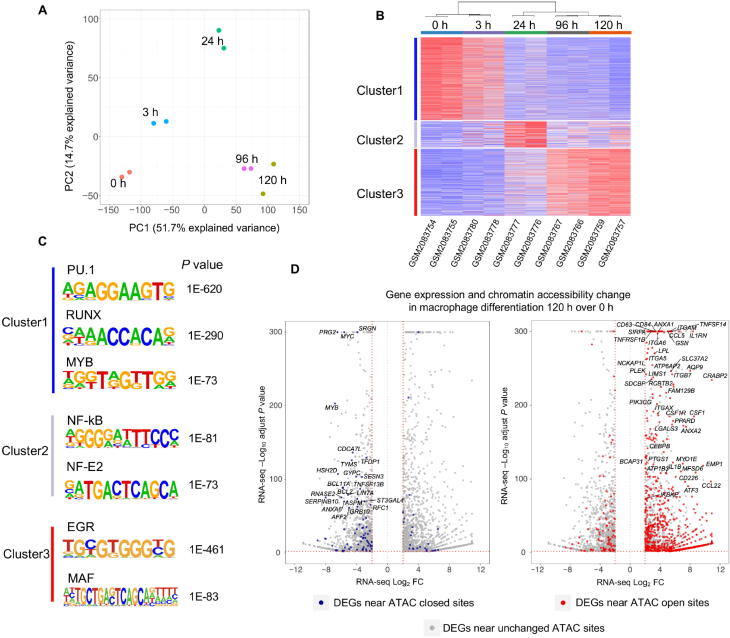
**Analysis of ATAC-seq data from HL-60 promyelocytes differentiating into macrophages with CoBRA. A.** PCA plot depicting how samples cluster along the first two principal axes. **B.** Sample–feature heatmap created by CoBRA. Sample clustering is shown on the horizontal axis and chromatin accessibility clustering is shown on the vertical axis. Clusters 1, 2, and 3 represent sites open at early, middle, and late differentiation stages, respectively. **C.** Transcription factor binding motifs enriched in early, middle, and late differentiation stages identified by CoBRA. **D.** Distribution of DEGs during macrophage differentiation (120 h over 0 h). DEGs that have nearby differential chromatin changes during differentiation are indicated with dots in red (open ATAC sites) or blue (closed ATAC sites), while DEGs near the unchanged ATAC sites are indicated with gray dots. DEG, differentially expressed gene.

In this example, we illustrate the efficacy of CoBRA analysis of ATAC-seq experiments by following the chromatin accessibility profile of differentiating cells [26]. In this experiment, researchers utilized a 5-day time course (0 h, 3 h, 24 h, 96 h, and 120 h) to profile accessible chromatin of HL-60 promyelocytes differentiating into macrophages (GEO: GSE79019). The CoBRA output includes a PCA plot (Figure 4**A**) that demonstrates the temporal differentiation of the macrophages, with the early time point on the left side and the late time point on the right. Furthermore, the output includes a sample–feature heatmap utilizing *k*-means (*k* = 3) clustering (Figure 4B) that further illustrates the dramatic differences in open chromatin profiles. The three clusters show clear differences in open chromatin between the early (Cluster 1), intermediate (Cluster 2), and late stage (Cluster 3) time points.

CoBRA automatically performs a *de novo* motif analysis on each of the three clusters of accessible sites to identify motifs of potential transcriptional regulators enriched in differentially accessible chromatin elements. This analysis identified many transcription factor binding motifs enriched in each cluster (Figure 4C). Motifs for PU.1, RUNX, and MYB are enriched in Cluster 1, which exhibits a decrease in accessibility during myeloid differentiation. It is likely that a depletion of PU.1, RUNX, and MYB occupancy occurs at these elements during cellular commitment. In addition, we observe the EGR and MAF motifs in Cluster 3, suggesting that a gain of EGR and MAF occurs at these elements during macrophage differentiation. The motif analysis for Cluster 2 also identified chromatin element NF-κB and NF-E2 as being active during differentiation and depleted in the latter stage. All of these findings are consistent with the results from published papers [26].

Finally, ChIP-seq and ATAC-seq data are often generated in parallel with RNA-seq data on the same samples. An extension to CoBRA can take the differential expression gene list from RNA-seq data analysis tools such as VIPER [27] and highlight differentially expressed genes that also exhibit differential chromatin accessibility. The volcano plot in Figure 4D is a visualization of the genes differentially expressed during macrophage differentiation and highlights the genes that also have nearby opening chromatin during differentiation. Expression of genes near open chromatin during differentiation is more likely to be upregulated. This profile that combines chromatin accessibility with gene expression can provide insight to potentially identify major transcriptomic elements driving differentiation.

## Discussion

The case studies that we have presented highlight typical use cases for CoBRA. The first example is accurate identification of differential peaks by appropriate normalization of ChIP-seq data. Some methods fail to normalize ChIP-seq data appropriately in calling differential peaks when the FRiP score is impacted by perturbations. CoBRA reduces false positives and identifies more true differential peaks by appropriately normalizing ChIP-seq data according to sequencing depth.

The second example demonstrates how CoBRA can be used to account for amplification due to CNVs present in experimental samples. This is an important feature, as CNVs can drive the greatest differences between some tumor samples and obscure other biological changes in the data available in Cistrome DB that occur as a result of treatment or other experimental conditions. After correction of CNVs, differential peaks called by DESeq2 will not be affected by amplification between samples, allowing biologists to better understand whether differences are caused by changes in the genetic or epigenetic landscapes.

The third example illustrates how CoBRA can be applied to ATAC-seq experiments. Unsupervised analyses can identify changes in the chromatin accessibility over time with treatment, and clustering provides insight into similarities and differences between samples, and aids the investigation of the transcription factor motif enrichment in each cluster.

The application of CoBRA to these experiments demonstrates the broad capabilities of the workflow in analyzing ChIP-seq or ATAC-seq experiments. While other workflows used to analyze ChIP-seq or ATAC-seq experiments are available, they lack some of the features present in CoBRA (Table 1). Additionally, the highly modular Snakemake framework allows for rapid integration of new approaches or replacement of existing tools. Modules can be added simply by adding a new Snakemake “rule” and adding a flag in the config file (Figure S1A–C) to turn the analysis on. Moreover, “rules” in CoBRA can be composed of tools written in R, Python, or shell script. The framework allows for great flexibility because each module can be evaluated in its own environment using different tools (*e.g.*, software based on Python 2.7 and Python 3).

**Table 1:** Comparison of features available in CoBRA with other pipelines

**Feature**	**CoBRA**	**DiffBind**	**HMCan-diff**	**ChIPComp**	**deepTools**	**esATAC**	**OPENANNO**
Sample–sample correlation	✓		✓		✓	✓	
Sample–feature clustering	✓	✓			✓		
PCA	✓	✓					
Normalization based on sequencing depth	✓	✓					
CNV correction for differential peak calling	✓		✓				
Motif analysis	✓					✓	
Pathway analysis	✓					✓	
Package easy update	✓	✓		✓		✓	✓
Easy support for new species	✓						
Docker containerized	✓						
Peak region annotation with public ChIP-seq databases	✓						✓
Step-by-step tutorial with multiple case studies	✓	✓			✓	✓	
Weblink	https://cfce-cobra.readthedocs.io/	https://bioconductor.org/packages/release/bioc/html/DiffBind.html	https://www.cbrc.kaust.edu.sa/hmcan/hmcan-diff_desc.php	https://bioconductor.org/packages/release/bioc/html/ChIPComp.html	https://deeptools.readthedocs.io/	https://www.bioconductor.org/packages/release/bioc/html/esATAC.html	http://health.tsinghua.edu.cn/openannotate/

*Note*: CoBRA, Containerized Bioinformatics workflow for Reproducible ChIP/ATAC-seq Analysis; CNV, copy number variation; PCA, principal component analysis.

The methods for installing, deploying, and using CoBRA along with a detailed tutorial are provided in the documentation available online (https://cfce-cobra.readthedocs.io/). The workflow was designed to work with Docker, which allows the user to automatically download all required software dependencies, eliminating the possibility of conflicting dependencies. This makes CoBRA easy for the user with limited computational training to install and run the workflow. Furthermore, the user does not need to prepare any reference files, as CoBRA automatically downloads all needed reference files. As a result, CoBRA is portable, reproducible, and easy to deploy. In summary, we have developed a new pipeline, CoBRA, that is fast, efficient, portable, customizable, and reproducible. The workflow is built upon the ongoing effort to make computational studies reproducible using defined workflows run inside Docker containers. CoBRA allows users with varying levels of computational skills to quickly process and analyze new data from ChIP-seq and ATAC-seq experiments. It is our hope that CoBRA can be a starting point for others to build upon and improve CoBRA as a tool and extend its ability to analyze the data in Cistrome.

## Code availability

CoBRA ia publicly available online at https://bitbucket.org/cfce/cobra and https://cfce-cobra.readthedocs.io.

## Competing interests

The authors have declared that no competing interests exist.

Available online 18 July 2021

Handled by Zhihua Zhang

2021 The Authors. Published by Elsevier B.V. and Science Press on behalf of Beijing Institute of Genomics, Chinese Academy of Sciences / China National Center for Bioinformation and Genetics Society of China.

This is an open access article under the CC BY license (http://creativecommons.org/licenses/by/4.0/).

### CRediT authorship contribution statement

**Xintao Qiu:** Methodology, Software, Validation, Writing – original draft. **Avery S. Feit:** Methodology, Software, Validation, Writing – original draft. **Ariel Feiglin:** Methodology, Software, Validation. **Yingtian Xie:** Validation, Data curation. **Nikolas Kesten:** Validation. **Len Taing:** Software, Validation. **Joseph Perkins:** Software. **Shengqing Gu:** Validation, Investigation. **Yihao Li:** Validation, Investigation. **Paloma Cejas:** Validation, Investigation. **Ningxuan Zhou:** Validation, Investigation. **Rinath Jeselsohn:** Resources, Validation. **Myles Brown:** Conceptualization, Supervision, Funding acquisition. **X. Shirley Liu:** Supervision, Project administration. **Henry W. Long:** Supervision, Funding acquisition, Conceptualization, Writing – review & editing.

## Supplementary material

Supplementary Figure S1 **Configuration file used by CoBRA**. **A**. Unsupervised parameters in config.yaml file. **B**. Supervised parameters in config.yaml file. **C**. Input file information in config.yaml file.

Supplementary Figure S2 **File structure of CoBRA input and output**. **A**. File and folders for input and output. **B**. Example of file structure inside the output folder -- analysis.

Supplementary Figure S3 **PCA and HOMER motif analyses of the GR dose-response dataset**. **A**. PCA plot depicting similarity between dexamethasone-treated samples in case study 1. **B**. Clustering result of HOMER motif enrichment analysis for differential GR binding sites enriched in samples treated with 50 nM dexamethasone compared to those treated with 0.5 nM dexamethasone. GR, glucocorticoid receptor; PCA, principal component analysis.
