# Supplementary material for: CoBRA: Containerized Bioinformatics Workflow for Reproducible ChIP/ATAC-seq Analysis
Source: Genomics Proteomics Bioinformatics. 2021 Jul 18;19(4):652–61. doi: 10.1016/j.gpb.2020.11.007 (PMC9039557; doi:10.1016/j.gpb.2020.11.007)
Supplement: Supplementary Figure S1 — Configuration file used by CoBRA. A. Unsupervised parameters in config.yaml file. B. Supervised parameters in config.yaml file. C. Input file information in config.yaml file. [file mmc1.pdf]

A Unsupervised parameters in config.yaml file

```yaml
#Project Name
#Use in pca, sample-sample, sample-feature plot. Please use "_" to seperate different words
project: ChIP_seq

#enhancer option enhancer/promoter/all
enhancer: all

#Location of metasheet
metasheet: metasheet.csv
ref: "scripts/ref.yaml"


#Assembly is needed when seperate enhancer/promoter, motif finding, nearyby gene
assembly: hg19


#At least mini_num_sample should have RPKM > rpkm_threshold
rpkm_threshold: 1
mini_num_sample: 0


#Scale method for the nomalize counts among samples
#z- z-score
#q- quantile-normalize
#l- log-transform
scale: q

#Fliter metric in feature selection
#sd- Standard deviation
#cov- Coefficient of Variation
#av- mean
filter-opt: cov

#top percent cutoff
filter-percent: 100

#limited of peaks to use for plot
SSpeaks: 20000000
SFpeaks: 20000000

#number of k-means clustering in sample-feature plot
num_kmeans_clust: 6

#correlation method for sample-sample, sample-feature plot
# "person" or "spearman"
cor_method: 'pearson'

#distance method for sample-sample, sample-feature plot
# "euclidean", "manhattan", "canberra", "binary", "maximum" or "minkowski"
dis_method: 'euclidean'
```

B Supervised parameters in config.yaml file

```yaml
#DEseq_cut_off - Padj/LG2FC
Padj: 0.05
LG2FC: 0

#DEseq normalize method
#def - normlize by default setting of DEseq2
#depth - normlize by the sequence depth of each sample
nor_method: 'depth'

#Motif analysis - true/false
motif: 'false'

#CNV correction? true/false
CNV_correction: 'false'

#unchanged heatmap
unchanged_heatmap: 'false'
```

C Input file information in config.yaml file

```yaml
#BAM files sorted? true/false
bam_sort: 'true'


#fastq as input
fastq_in: 'true'

#number of threads used in bwa mem
thread: 8


# sample names, e.g. "sample01" "sample02" can be any abitrary string
# HOWEVER, these names must match what is in metasheet.csv
# FOR each sample, define the path to the fastq file

fastq:
   sample1:
      - ./XX1_R1.fastq.gz
   sample2:
      - ./XX2_R1.fastq.gz
   input:
      - ./XX_input_R1.fastq.gz


# bed, bam and bigwig is not needed when fastq_in is true
bed:
   sample1: ./XX1.bed
   sample2: ./XX2.bed

bam:
   sample1: ./XX1.bam
   sample2: ./XX2.bam


bigwig:
   sample1: ./XX1.bw
   sample2: ./XX2.bw

# tab-separated cnv files
cnv:
   sample1: ./XX1.igv
   sample2: ./XX2.igv
```
