# Supplementary material for: CoBRA: Containerized Bioinformatics Workflow for Reproducible ChIP/ATAC-seq Analysis
Source: Genomics Proteomics Bioinformatics. 2021 Jul 18;19(4):652–61. doi: 10.1016/j.gpb.2020.11.007 (PMC9039557; doi:10.1016/j.gpb.2020.11.007)
Supplement: Supplementary Figure S2 — File structure of CoBRA input and output. A. File and folders for input and output. B. Example of file structure inside the output folder -- analysis. [file mmc2.pdf]

Overview of CoBRA layout

A

metasheet.csv	Comma Separated Spreadsheet (.csv)
config.yaml	YAML
Snakefile	File
README.md	Markdown File
scripts	Directory
ref_files	Directory
analysis	Directory

B

- analysis
  - clustering_analysis
    - rpkm.1_num_sample.0_scale.q_fliter.cov.2
      - plots
        - images
          - heatmapSF_plot_2_percent
          - heatmapSS_plot_2_percent
          - pca_plot_2_percent
      - read_counts
    - rpkm.2_num_sample.3_scale.q_fliter.cov.2
  - differential_peaks
    - Met_over_Tumor
      - GSEA
        - C2_Curated.GseaPreranked.1587247351823
        - H_Hallmark.GseaPreranked.1587247292750
      - cistrome_toolkit
  - logs
    - clean_bam
    - read_coutns
    - remove_duplicates
    - reorder
  - preprocessed_files
    - bam
      - clean_bam
      - remove_chrMY
      - remove_duplicates
      - reorder
      - sorted_reads
    - bed
    - bigwig
    - read_counts
