# Supplementary material for: CoBRA: Containerized Bioinformatics Workflow for Reproducible ChIP/ATAC-seq Analysis
Source: Genomics Proteomics Bioinformatics. 2021 Jul 18;19(4):652–61. doi: 10.1016/j.gpb.2020.11.007 (PMC9039557; doi:10.1016/j.gpb.2020.11.007)
Supplement: Supplementary Figure S3 — PCA and HOMER motif analyses of the GR dose-response dataset. A. PCA plot depicting similarity between dexamethasone-treated samples in case study 1. B. Clustering result of HOMER motif enrichment analysis for differential GR binding sites enriched in samples treated with 50 nM dexamethasone compared to those treated with 0.5 nM dexamethasone. GR, glucocorticoid receptor; PCA, principal component analysis. [file mmc3.pdf]

A

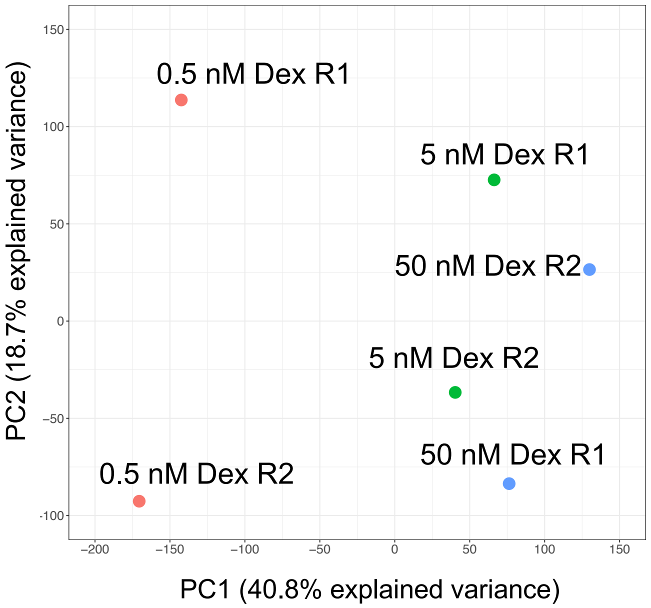

B

Cluster	Motif	Name	*P* Value	$Log_{10}(P\ Value)$	# Target sequences with motif	% of Target sequences with motif	# Background sequences with motif	% of Background sequences with motif
1	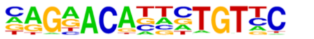	GRE(NR),IR3/RAW264.7-GRE-ChIP-Seq(Unpublished)/Homer	1e-2378	-5477.435464	3019.0	44.27%	1521.0	3.63%
	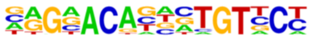	GRE(NR),IR3/A549-GR-ChIP-Seq(GSE32465)/Homer	1e-2142	-4932.236126	2349.0	34.44%	824.5	1.97%
	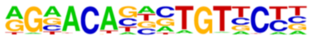	ARE(NR)/LNCAP-AR-ChIP-Seq(GSE27824)/Homer	1e-2031	-4677.025589	2876.0	42.17%	1742.3	4.16%
	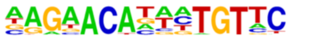	PGR(NR)/EndoStromal-PGR-ChIP-Seq(GSE69539)/Homer	1e-1835	-4225.926702	2602.0	38.15%	1540.2	3.67%
	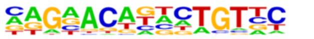	PR(NR)/T47D-PR-ChIP-Seq(GSE31130)/Homer	1e-1225	-2820.686095	5632.0	82.58%	16086.6	38.37%
2	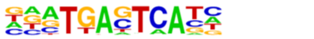	Fra1(bZIP)/BT549-Fra1-ChIP-Seq(GSE46166)/Homer	1e-1129	-2599.846466	2798.0	41.03%	3627.8	8.65%
	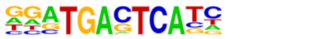	Fra2(bZIP)/Striatum-Fra2-ChIP-Seq(GSE43429)/Homer	1e-1093	-2518.963525	2590.0	37.98%	3139.8	7.49%
	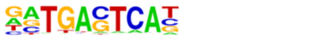	JunB(bZIP)/DendriticCells-Junb-ChIP-Seq(GSE36099)/Homer	1e-1040	-2396.976282	2728.0	40.00%	3729.9	8.90%
	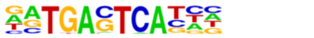	Atf3(bZIP)/GBM-ATF3-ChIP-Seq(GSE33912)/Homer	1e-1037	-2389.268204	2975.0	43.62%	4536.5	10.82%
	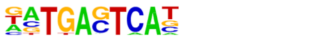	BATF(bZIP)/Th17-BATF-ChIP-Seq(GSE39756)/Homer	1e-1027	-2365.228649	2920.0	42.82%	4396.5	10.49%
	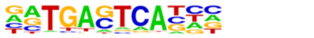	Fosl2(bZIP)/3T3L1-Fosl2-ChIP-Seq(GSE56872)/Homer	1e-1015	-2338.547207	2128.0	31.20%	2151.7	5.13%
	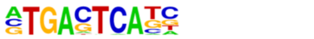	AP-1(bZIP)/ThioMac-PU.1-ChIP-Seq(GSE21512)/Homer	1e-950	-2188.543174	3045.0	44.65%	5166.9	12.32%
	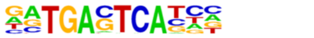	Jun-AP1(bZIP)/K562-cJun-ChIP-Seq(GSE31477)/Homer	1e-918	-2115.098968	1762.0	25.84%	1544.5	3.68%
	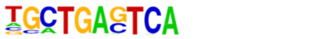	Bach2(bZIP)/OCILy7-Bach2-ChIP-Seq(GSE44420)/Homer	1e-493	-1135.631294	1172.0	17.18%	1285.2	3.07%
	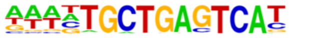	Bach1(bZIP)/K562-Bach1-ChIP-Seq(GSE31477)/Homer	1e-191	-440.281436	392.0	5.75%	343.5	0.82%
	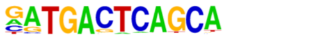	NF-E2(bZIP)/K562-NFE2-ChIP-Seq(GSE31477)/Homer	1e-189	-435.587020	427.0	6.26%	422.3	1.01%
	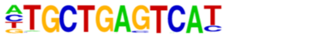	Nrf2(bZIP)/Lymphoblast-Nrf2-ChIP-Seq(GSE37589)/Homer	1e-168	-387.840346	362.0	5.31%	336.6	0.80%
	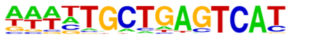	NFE2L2(bZIP)/HepG2-NFE2L2-ChIP-Seq(Encode)/Homer	1e-141	-325.917248	322.0	4.72%	320.6	0.76%
	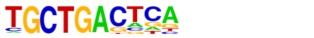	MafA(bZIP)/Islet-MafA-ChIP-Seq(GSE30298)/Homer	1e-76	-175.974922	1475.0	21.63%	5620.6	13.41%
